# Veterinary Medical Association of the District of Columbia

**Published:** 1897-10

**Authors:** J. P. Turner

**Affiliations:** Secretary


### VETERINARY MEDICAL ASSOCIATION OF THE DISTRICT OF COLUMBIA.

THE September meeting was held in Elks' Hall, 1006 E Street, N. W., on Saturday, the 25th, at 8 P.M., with the Vice-president, Dr. Buckingham, in the chair. Minutes of previous meeting approved.

A letter was presented from the Commissioners of the District, in which they stated that the application of this Society relative to the muzzling of dogs running at large during the summer months had been referred to the Attorney for the District, who gave his opinion that it was illegal to issue such an order. The Association directed the Secretary to call the attention of the commissioners to the police regulations which directly applied to the muzzling of dogs.

The Legislative Committee reported that at the next meeting it would propose a new paragraph for the bill which is to be introduced during the next session of Congress, regulating the practice of veterinary medicine and surgery in the District. This paragraph will be of a reciprocative nature, and will provide that such States as will in the future recognize the certificates granted by the Board of Veterinary Examiners, to be created by Congress in said bill, as entitling holders of said certificates to practise in their respective State without undergoing another examination by the State Board of Veterinary Examiners of said State, will be treated in a similar manner by the authorities of the District of Columbia, and veterinarians of such States will be privileged to practise in the District of Columbia, provided they have a certificate from a State Board of Veterinary Examiners.

Dr. C. B. Robinson moved that this Association extend invitation to the U. S. V. M. A. to hold its next meeting in this city; approved unanimously with cheers. Letters of regret were received from several members, among which was one from Dr. D. E. Salmon, recently elected a member of this Association, but promising to be more punctual in the future.

The regular subject of this meeting, which was the discussion of tuberculosis and its relations to the health and welfare of the District, was brought forward by Dr. C. B. Robinson presenting the following resolutions, which were unanimously adopted after a long discussion, and the Secretary was directed to forward them, along with a letter, to the Commissioners of the District:

*Whereas*, tuberculosis exists to a large extent among the cattle of the District of Columbia, Maryland and Virginia, all of which sections supply meat and milk to the city of Washington, D. C.;

*Whereas*, good beef, free of disease, is plentiful and comparatively cheap in this country;

*Whereas*, the slaughter for meat of cows which have been condemned for dairy purposes on account of tuberculosis and other diseases is constantly occurring in the abattoirs of the District and surrounding country; therefore, be it

*Resolved*, that it is the sense of this Association that such meat is extremely dangerous for human consumption, and that no cows which have been used in dairies should be allowed to be slaughtered for food except under the inspection of a veterinary surgeon in the employ of the District of Columbia, and that a copy of these resolutions be forwarded to the Commissioners of the District of Columbia.

J. P. TURNER,
Secretary.

